# Pellino-1 promotes lung carcinogenesis via the stabilization of Slug and Snail through K63-mediated polyubiquitination

**DOI:** 10.1038/cdd.2016.143

**Published:** 2016-12-23

**Authors:** Yoon Kyung Jeon, Chung Kwon Kim, Kyung Rim Hwang, Hye-Young Park, Jaemoon Koh, Doo Hyun Chung, Chang-Woo Lee, Geun-Hyoung Ha

**Affiliations:** 1 Department of Pathology, Seoul National University Hospital, Seoul National University College of Medicine, Seoul, Republic of Korea; 2 Department of Molecular Cell Biology, Samsung Biomedical Research Institute, Sungkyunkwan University School of Medicine, Suwon, Republic of Korea; 3 Department of Biomedical Sciences, Seoul National University College of Medicine, Seoul, Republic of Korea; 4 Department of Health Sciences and Technology, Samsung Advanced Institute for Health Sciences and Technology, Sungkyunkwan University, Suwon, Republic of Korea

## Abstract

Pellino-1 is an E3 ubiquitin ligase acting as a critical mediator for a variety of immune receptor signaling pathways, including Toll-like receptors, interleukin-1 receptor and T-cell receptors. We recently showed that the Pellino-1-transgenic (Tg) mice developed multiple tumors with different subtypes in hematolymphoid and solid organs. However, the molecular mechanism underlying the oncogenic role of Pellino-1 in solid tumors remains unknown. Pellino-1-Tg mice developed adenocarcinoma in the lungs, and Pellino-1 expression was higher in human lung adenocarcinoma cell lines compared with non-neoplastic bronchial epithelial cell lines. Pellino-1 overexpression increased the cell proliferation, survival, colony formation, invasion and migration of lung adenocarcinoma cells, whereas Pellino-1 knock-down showed the opposite effect. Pellino-1 overexpression activated PI3K/Akt and ERK signaling pathways and elicited an epithelial–mesenchymal transition (EMT) phenotype of lung adenocarcinoma cells. Pellino-1-mediated EMT was demonstrated through morphology, the upregulation of Vimentin, Slug and Snail expression and the downregulation of E-cadherin and *β*-catenin expression. Notably, Pellino-1 had a direct effect on the overexpression of Snail and Slug through Lys63-mediated polyubiquitination and the subsequent stabilization of these proteins. Pellino-1 expression level was significantly correlated with Snail and Slug expression in human lung adenocarcinoma tissues, and lung tumors from Pellino-1-Tg mice showed Snail and Slug overexpression. The Pellino-1-mediated increase in the migration of lung adenocarcinoma cells was mediated by Snail and Slug expression. Taken together, these results show that Pellino-1 contributes to lung tumorigenesis by inducing overexpression of Snail and Slug and promoting EMT. Pellino-1 might be a potential therapeutic target for lung cancer.

Epithelial–mesenchymal transition (EMT) has important roles in the formation of the body plan and in the differentiation of various tissues and organs.^1, 2^ Moreover, EMT has a critical role in the carcinogenic process through various molecular pathways.^1, 3, 4^ EMT promotes tumor cell invasion and metastasis, leading to the generation of cancer cells with stem cell-like characteristics and resistance to chemotherapy.^4, 5^ The loss of expression of the epithelial marker E-cadherin is a hallmark of EMT, associated with the upregulation of E-cadherin transcriptional repressors, such as Snail, Slug, Twist, ZEB1 and ZEB2.^6, 7, 8, 9^ In addition, EMT is regulated through various signaling networks including ERK, MAPK, PI3K/Akt, TGF-*β*, Wnt/*β*-catenin and Notch pathways.^2, 4, 10, 11, 12^

Lung cancer remains the leading cause of cancer-related incidence and mortality worldwide.^13, 14^ Previous studies have suggested a role for EMT in lung cancer, although the molecular mechanism driving EMT remains elusive. The loss of E-cadherin through genetic or epigenetic alterations is correlated with the aggressive behavior of lung cancer.^4, 15, 16^ Moreover, transcriptional repressors of E-cadherin are upregulated in lung cancer, associated with tumor progression and poor prognosis of patients.^17, 18, 19, 20^

The Pellino family comprises three evolutionally conserved members (Pellino-1/2/3) that possess Ring-like motifs, a defining feature of the RING class of E3 ubiquitin ligases.^21^ Pellino proteins interact with various E2 enzymes to form Lys11-, Lys48- and Lys63-linked polyubiquitin chains *in vitro* and *in vivo*.^22, 23, 24^ Recent studies have demonstrated that Pellino-1 regulates innate and adaptive immune signaling pathways, including Toll-like receptors (TLRs), interleukin-1 receptor (IL-1R) and T-cell receptor signaling,^25, 26^ and also involves in regulating the proliferation and activation of B and T cells. However, the biological role of Pellino in tumorigenesis remains unclear. Recent studies have indicated that Pellino-1 activates NF-κB and MAPK signaling pathways in human and murine immune cells.^27, 28, 29^ Interestingly, we previously used transgenic (Tg) mice to assess the gain of function for Pellino-1 and observed the increased incidence of tumors in the lungs, livers, lymph nodes, and other organs of Pellino-1-Tg mice.^30^ This observation suggested that Pellino-1 might contribute to the tumor development of different types.^30^ Moreover, we provided the first evidence that Pellino-1 is a novel oncogene contributing to B-cell lymphomagenesis through induction of BCL6 via Lys(K)63-mediated polyubiquitination and consequent stabilization.^30^ However, the mechanism by which Pellino-1 promotes development of epithelial tumor in lungs remains unknown. Thus, we addressed this issue here.

In this study, we demonstrated a novel mechanism through which Pellino-1 contributes to lung tumorigenesis by promoting EMT. The results showed that the Pellino-1-mediated ubiquitination and overexpression of Slug and Snail promotes EMT in lung cancer.

## Results

### Aberrant overexpression of Pellino-1 promotes lung cancer development

**Figure 1:**
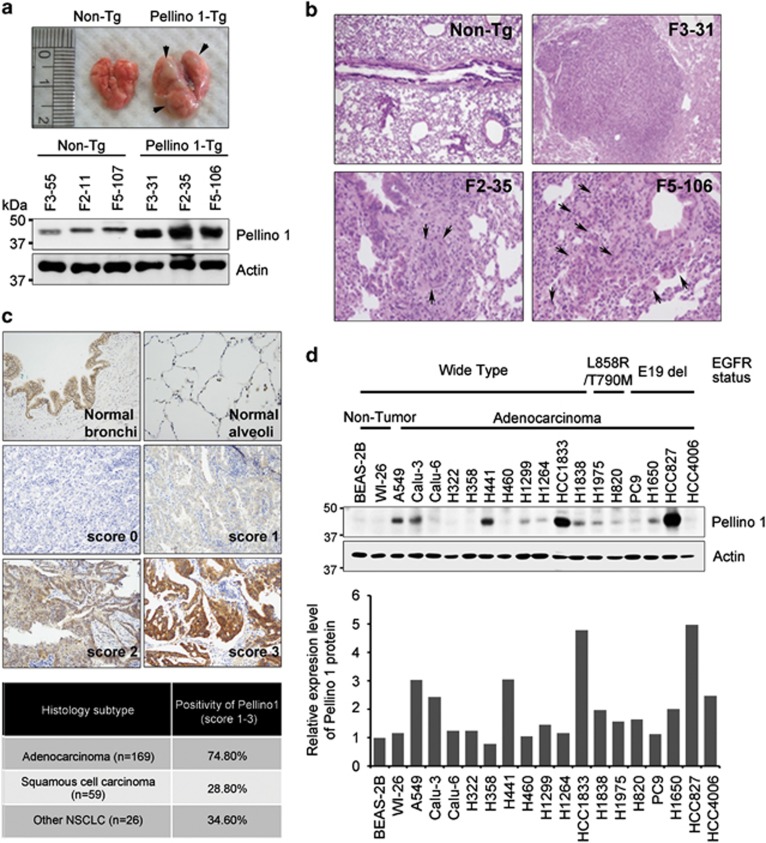
Pellino-1-Tg mice develop lung cancer and Pellino-1 is overexpressed in human lung cancers. (**a**) Representative macroscopic images showing lung tumors (arrows) in Pellino-1-Tg mice (top). Lysates of non-Tg and Pellino-1-Tg lung tissues were subjected to immunoblotting for Pellino-1 and actin (bottom). (**b**) Microscopic images for lung adenocarcinoma (F3-31), dysplasia (F2-35) and AAH-like lesions (F5-106) developed in Pellino-1-Tg mice. (**c**) Representative IHC images for Pellino-1 in normal human lung tissues (upper) and lung cancers (middle and lower) (original magnification, × 400). Pellino-1 expression rate was analyzed according to the histological subtype of human lung cancer including adenocarcinoma, squamous cell carcinoma and other NSCLC histology (bottom table). (**d**) Two non-tumor human lung cell lines (BEAS-2B and WI-26) and 17 human lung cancer cell lines (all adenocarcinomas except for H460) with WT EGFR or mutated EGFR were analyzed for Pellino-1 expression using immunoblotting (top). Pellino-1 expression level was quantified through scanning densitometry with actin as an internal control (bottom)

Multiple tumors were observed in the lungs of Pellino-1-Tg mice, but not in non-Tg littermates (Figure 1a). Pellino-1 expression was much higher in the lung tissues of Pellino-1-Tg mice compared with non-Tg mice (Figure 1a). Histopathological examination of the lungs of Pellino-1-Tg mice showed adenocarcinoma (F3-31), dysplasia (F2-35) and atypical adenomatous hyperplasia (AAH)-like lesions (F5-106) (Figure 1b).

In non-neoplastic human lungs, Pellino-1 was highly expressed in bronchial epithelial cells, moderately expressed in alveolar macrophages and weakly expressed in alveolar pneumocytes (Figure 1c, upper). Pellino-1 was variably expressed in human lung cancer tissues (Figure 1c, middle) with the highest frequency in adenocarcinoma (Figure 1c, lower) among non-small cell lung cancer (NSCLC). Pellino-1 expression was evaluated in an extended cohort of pulmonary adenocarcinoma (*n*=491). Pellino-1 overexpression was significantly higher in patients with adenocarcinoma harboring EGFR mutation, increased EGFR gene copy numbers and MET expression (Supplementary Table S1). However, Pellino-1 expression was not an independent prognostic factor in multivariate analysis for disease-free survival after surgical resection of the tumor (data not shown). Pellino-1 expression was observed up to 65% (11/17) of lung adenocarcinoma cell lines with a relative value of expression exceeding 1.5 compared with non-neoplastic lung cell lines (BEAS-2B and WI-26) (Figure 1d). Together, these data suggest that the overexpression of Pellino-1 might be involved in the development of lung adenocarcinoma.

### Pellino-1 promotes proliferation, survival, migration, invasion and oncogenic transformation in lung cancer cells

**Figure 2:**
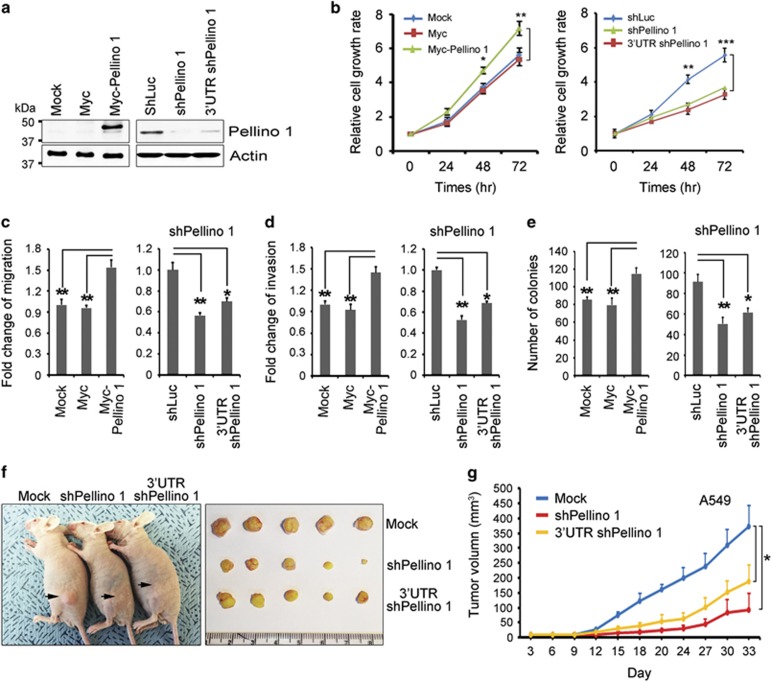
Pellino-1 overexpression enhances the cellular proliferation, migration, invasion and oncogenic transformation in A549 cells. (**a**) A549 cells were transfected with Myc (control), Myc-tagged Pellino-1, shLuc (control), Pellino-1-targeted shRNA (shPellino-1) or 3′-UTR Pellino-1-targeted shRNA (3′-UTR shPellino-1), and subjected to immunoblotting for Pellino-1 and actin. (**b**) MTT assay was performed to estimate the cell proliferation of A549 cells with Pellino-1 overexpression or knock-down. (**c** and **d**) A549 cells with Pellino-1 overexpression (Myc-Pellino-1) or Pellino-1 depletion (shPellino-1 and 3′-UTR shPellino-1) were pretreated with 10 *μ*g/ml mitomycin C for 1 h at 37 °C, washed twice with PBS and then were subjected to transwell migration (**c**) and invasion assays (**d**). (**e**) A549 cells with Pellino-1 overexpression or Pellino-1 depletion were subjected to colony-forming assay. The numbers of colonies were counted in four randomly selected microscopic fields per plates. (**f-g**) A549 cells (5 × 10^6^) transfected with shLuc, shPellino-1 or 3′-UTR shPellino-1 were inoculated subcutaneously into the flank of athymic nude mice (five mice per group). Animals were monitored up to 33 days and tumor size was measured using an electronic caliper at 3-day intervals. All data are shown as the means±S.D. of at least three independent experiments. The *P-*values were calculated using unpaired Student's *t*-test. *P <0.05; **P <0.01; ***P <0.005

To evaluate the functional significance of Pellino-1 in lung tumorigenesis, Pellino-1 in A549 cells was overexpressed or knock-downed by transfection with Myc-Pellino-1, shPellino-1 or 3′-untranslated region (UTR) shPellino-1 (Figure 2a). The overexpression of Pellino-1 in A549 cells increased cell proliferation, whereas the depletion of Pellino-1 reduced cell proliferation (Figure 2b). The overexpression of Pellino-1 enhanced cell migration and invasion, whereas the depletion of Pellino-1 suppressed the migration and invasion of A549 cells (Figures 2c and d). Similar results were observed in Pellino-1-overexpressing or knocked-down H1299 cells as well as in Pellino-1-overexpressing BEAS-2B cells (Supplementary Figures S1 and S2). The overexpression of Pellino-1 promoted anchorage-independent growth and colony-forming ability, whereas the depletion of Pellino-1 resulted in the opposite effect in A549 cells (Figure 2e). Moreover, in *in vivo* xenograft model, tumor growth was significantly decreased in mice injected with Pellino-1 knocked-down A549 cells compared with control (Figures 2f and g). In contrast, Pellino-2 and -3 (*α* and *β*) had no influence on the cell proliferation, survival, migration and invasion in A549 cells (Supplementary Figures S3a-d). Together, these data suggest that Pellino-1 promotes the proliferation, migration, invasion, tumor growth and oncogenic transformation of lung cancer cells.

### Pellino-1 promotes the EMT and activates PI3K/Akt and ERK signaling pathways in lung cancer cells

**Figure 3:**
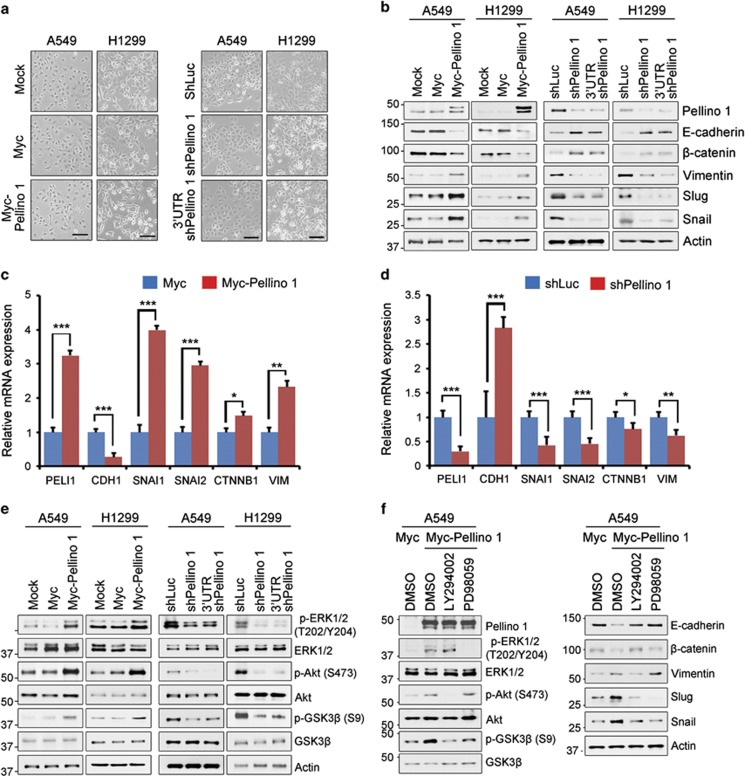
Pellino-1 promotes the EMT phenotype and activates the PI3K/Akt and ERK signaling pathways. (**a**) Representative phase-contrast images for Pellino-1-overexpressing A549 and H1299 cells (left) and Pellino-1-depleted A549 and H1299 (right). The scale bars represent 100 *μ*m. (**b**) A549 and H1299 cells were transfected with the Myc or Myc-Pellino-1 (left), and the shLuc, shPellino-1 or 3′-UTR shPellino-1 (right), and subjected to immunoblotting for Pellino-1, E-cadherin, *β*-catenin, Vimentin, Slug, Snail and actin. (**c** and **d**) Analysis of *PELI1* (encoding to Pellino-1), *CDH1* (encoding to E-cadherin), *SNAI1* (encoding to Snail), *SNAI2* (encoding to Slug), *CTNNB1* (encoding to *β*-catenin), *VIM* (encoding to Vimentin) mRNA levels was performed by qRT-PCR in Pellino-1-overexpressing (**c**) and -knocked-down (**d**) A549 cells. (**e**) A549 and H1299 cells were transfected with the Myc or Myc-Pellino-1 (left), and the shLuc, shPellino-1 or 3′-UTR shPellino-1 (right), and subjected to immunoblotting for p-ERK1/2 (T202/Y204), ERK1/2, p-Akt (S473), Akt, p-GSK3*β* (S9), GSK3*β* and actin. (**f**) A549 cells were transfected with the Myc or Myc-Pellino-1, and Pellino-1-overexpressing A549 cells were subsequently treated with LY294002 (20 *μ*M) or PD98059 (20 *μ*M). After incubation of 12 h, the cells were lysed and subjected to immunoblotting with the indicated antibodies. All data are shown as the means±S.D. of at least three independent experiments. The *P*-values were calculated using unpaired Student's *t-*test. *P <0.05; **P <0.01; ***P <0.005

Of note, Pellino-1-overexpressing A549 and H1299 cells displayed stretched or elongated spindle cell morphology, whereas the depletion of Pellino-1 increased cell-to-cell contacts (Figure 3a). Thus, it was hypothesized that EMT might be involved in Pellino-1-mediated lung tumorigenesis. Consistent with morphological changes, the overexpression of Pellino-1 reduced the expression of epithelial markers, including E-cadherin and *β*-catenin, but increased the expression of mesenchymal markers, including Vimentin, Snail, and Slug, in A549 and H1299 cells (Figure 3b, left). No significant changes were observed in Twist and ZEB1 expression (data not shown). Moreover, the depletion of Pellino-1 reduced the expression of Vimentin, Snail and Slug but increased the E-cadherin and *β*-catenin expression (Figure 3b, right). The expression of E-cadherin is negatively regulated by transcription factor, such as Snail, Slug, Twist and ZEB1.^31, 32, 33^ Both protein and mRNA expression of Snail and Slug were markedly increased in Pellino-1-overexpressing cells (Figures 3b and c) and decreased in Pellino-1-depleted cells (Figures 3b and d) compared with control cells. In contrast, E-cadherin expression was markedly decreased in Pellino-1-overexpressing cells (Figures 3b and c) and increased in Pellino-1-depleted cells (Figures 3b and d) at both protein and mRNA levels. These findings suggest that Pellino-1 has an important role in the progression of EMT through the upregulation of E-cadherin repressors, such as Snail and Slug.

Cell survival and EMT are associated with activation of PI3K/Akt and MAPK/ERK pathways.^34^ PI3K/Akt activation phosphorylates and inactivates GSK3*β* and thus inhibits active GSK3*β* (i.e., dephosphorylated form)-induced degradation of Snail protein.^35, 36^ Thus, it was examined whether Pellino-1 regulates these signaling pathways. The phosphorylation of Akt, ERK1/2 and GSK3*β* was elevated in Pellino-1-overexpressing A549 and H1299 cells (Figure 3e, left), but decreased in Pellino-1-depleted A549 and H1299 cells (Figure 3e, right). In Pellino-1-overexpressing A549 cells, LY294002 (PI3K inhibitor) and PD98059 (MEK inhibitor) reduced the Snail and Slug expression but increased the E-cadherin expression (Figure 3f). Overexpression of GSK3*β* significantly reduced the levels of Slug and Snail as previously reported.^35, 37^ Thus, it was determined whether overexpression of Pellino-1 enhances the stability of Snail and Slug proteins in GSK3*β*-overexpressing cells. GSK3*β* overexpression in A549 cells reduced the Snail and Slug expression, whereas the overexpression of Pellino-1 in the GSK3*β*-overexpressing cells significantly recovered the levels of Slug and Snail (Supplementary Figure S4). This data suggest that Pellino-1 might prevent the GSK3*β*-mediated Slug and Snail degradation. Taken together, these results indicated that Pellino-1-mediated activation of PI3K/Akt and ERK signaling pathways might be implicated in Pellino-1-mediated cell proliferation and EMT progression.

### Pellino-1 interacts and regulates Slug and Snail stabilization via K63-mediated ubiquitination

**Figure 4:**
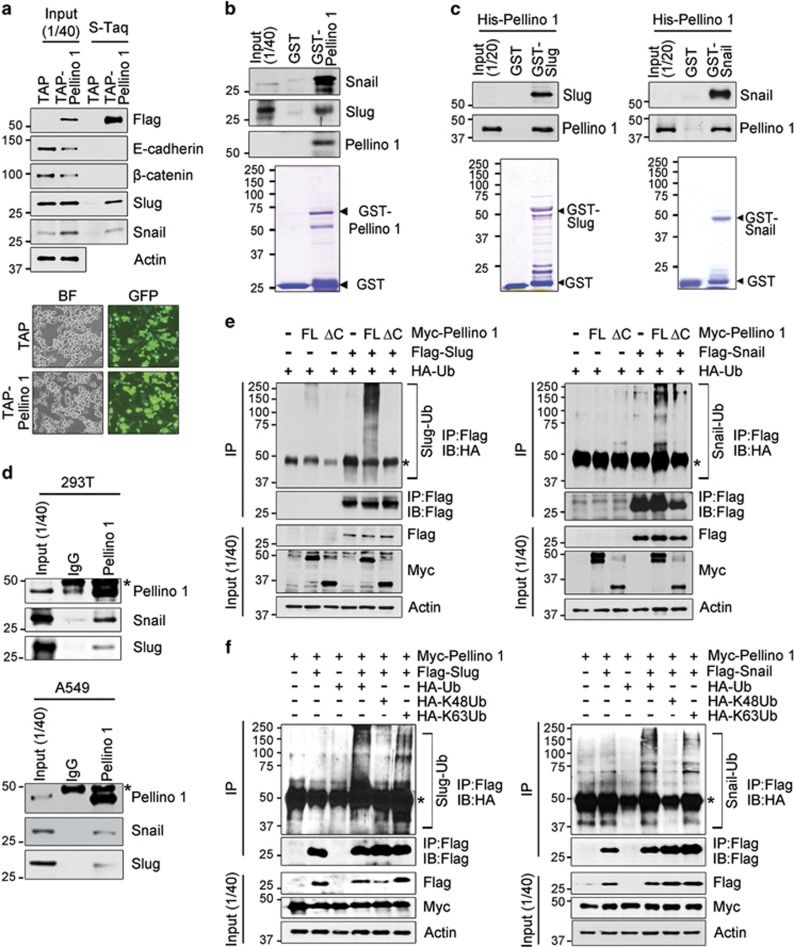
Pellino-1 directly interacts with Slug and Snail, and promotes K63-mediated polyubiquitination of Slug and Snail. (**a**) 293T cells were transfected with TAP (control), TAP-Pellino-1 (Flag-tagging Pellino-1). At 36 h post-transfection, the cells were inspected for GFP expression under microscope (lower) and harvested and isolated through tandem affinity purification (TAP). The bound proteins were immunoblotted with the indicated antibodies (upper). Transfection efficiency was evaluated by inspecting cells expressing GFP under microscope (BF, bight filed; lower). **(b)** A549 cell lysates were incubated with GST or GST-Pellino-1, and subjected to immunoblotting with the indicated antibodies. (**c**) GST, GST–Slug or GST–Snail fusion proteins were incubated with purified His-Pellino-1, and subjected to immunoblotting for Slug, Snail and Pellino-1. (**d**) 293T (upper panel) or A549 (bottom panel) cell lysates were immunoprecipitated with anti-Pellino-1 antibody and immunoblotted with anti-Snail, anti-Slug and anti-Pellino-1 antibodies. Asterisk denotes nonspecific band. (**e**) HCT116 cells were transfected with Myc-Pellino-1 FL or Myc-Pellino-1-ΔC (ring domain deletion mutant) in combination with HA-Ub and Flag-Slug (left) or Flag-Snail (right). At 36 h post-transfection, the cells were harvested and immunoprecipitated with an anti-Flag antibody. The Slug or Snail protein complexes were subjected to immunoblotting with an anti-HA and anti-Flag antibodies. (**f**) HCT116 cells were transfected with Myc-Pellino-1, HA-UB, HA-UB K48, HA-Ub K63 and Flag-Slug (left) or Flag-Snail (right) in combination. At 36 h post-transfection, the cells were harvested and immunoprecipitated with an anti-Flag antibody. The Slug or Snail protein complexes were subjected to immunoblotting with anti-HA and anti-Flag antibodies

Pellino-1 is an E3 ligase. Thus, it was hypothesized that Pellino-1 might interact with Slug and Snail to regulate their stabilities. Extracts from asynchronously growing TAP and TAP-Pellino-1 (Flag-tagged Pellino-1) transfected 293T cells were subjected to pull-down assays (Figure 4a), which showed Pellino-1 interacts with endogenous Snail or Slug but not with E-cadherin and *β*-catenin. Next, GST–Pellino-1 fusion protein was incubated with cellular extracts from asynchronized A549 cells (Figure 4b), and GST–Slug or GST–Snail fusion proteins were incubated with purified His-tagged Pellino-1 (Figure 4c). Immunoprecipitation assay for Pellino-1 and Snail or Slug was also performed in 293T and A549 cells (Figure 4d). Immunoprecipitation assay was also performed using GFP fusion Pellino-1 full-length (FL), ring-like domain deleted (ΔC) and ring-like domain only (C) plasmids to see interactions with Snail and Slug (Supplementary Figure S5). Results from these assays suggested that Pellino-1 interacts with Snail and Slug via FHA domain of Pellino-1.

Of note, the overexpression of FL Pellino-1 (Pellino-1-FL) increased the expression of Slug and Snail in A549 cells, but the overexpression of Pellino-1 C-terminal RING domain deletion mutant (Pellino-1-ΔC), which has defect in E3 ligase activity, did not (Supplementary Figures S6a and b). The Akt and ERK1/2 phosphorylation and cell migration was increased by Pellino-1-FL in A549 cells, but not by Pellino-1-ΔC (Supplementary Figures S6b-c). These data suggested that Pellino-1's function in A549 cells might depend on its E3 ligase activity. Thus, ubiquitination status of Slug and Snail affected by Pellino-1 was evaluated. To this end, HCT116 cells were transfected with Myc-Pellino-1-FL or Myc-Pellino-1-ΔC in combination with HA-Ub and Flag-Slug or Flag-Snail, and subjected to immunoprecipitation with an anti-Flag antibody and subsequent immunoblotting. Interestingly, the polyubiquitinated forms of Slug (Figure 4e, left) and Snail (Figure 4e, right) were evident in cells transfected with Pellino-1-FL, but not in cells transfected with Pellino-1-ΔC. To further examine the ubiquitination site of Slug and Snail through Pellino-1, HCT116 cells were transfected with Myc-Pellino-1 in combination with HA-Ub, HA-K48Ub, HA-K63Ub, Flag-Slug or Flag-Snail, and analyzed as above (Figure 4f). The overexpression of Pellino-1 increased the amount of HA-Ub and HA-Ub K63 polyubiquitinated forms of Slug and Snail, but not the HA-Ub K48 polyubiquitinated forms. In addition, endogenous polyubiquitinated forms of Snail and Slug proteins was evaluated in A549 cells transfected with TAP and TAP-Pellino-1 combined with His-Ub (Supplementary Figure S7). Overexpression of Pellino-1 (TAP-Pellino-1) increased the endogenous polyubiquitinated forms of Snail and Slug proteins compared with control cells (TAP) (Supplementary Figure S7). Together, these results indicate that Pellino-1 might directly regulate Slug and Snail via Lys(K)63-mediated polyubiquitination.

### Pellino-1 expression promotes Slug and Snail stabilization in lung cancer cells

**Figure 5:**
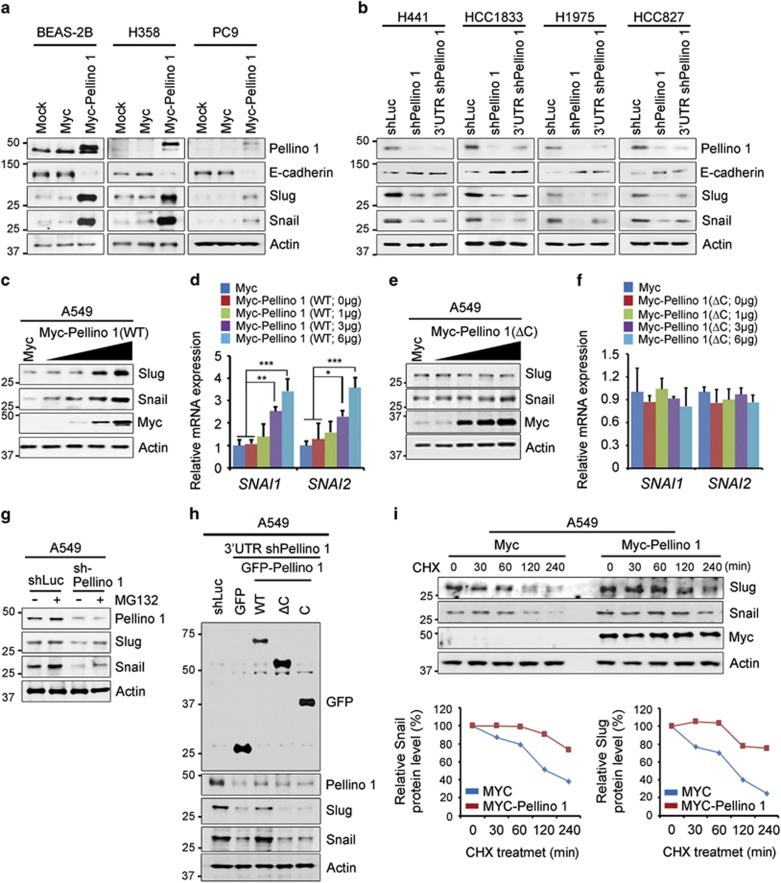
Pellino-1 promotes Slug and Snail stabilization in lung cancer cells. (**a**) BEAS-2B, H358 and PC9 cells (Pellino-1^Low^) were transfected with Myc or Myc-Pellino-1. At 48 h post-transfection, the cells were harvested and subjected to immunoblotting for Pellino-1, E-cadhrin, Snail, Slug and actin. (**b**) H441, H1833, H1975 and HCC827 cells (Pellino-1^High^) were transfected with shLuc, shPellino-1 or 3′-UTR shPellino-1. At 48 h post-transfection, the cells were harvested and subjected to immunoblotting for Pellino-1, E-cadhrin, Snail, Slug and actin. (**c**) A549 cells were transfected with Myc or Myc-Pellino-1 (WT; 0, 1, 3, 6* μ*g of plasmid). At 48 h post-transfection, the cells were harvested and subjected to immunoblotting for Myc, Snail, Slug and actin and (**d**) qRT-PCR analysis for *SNAI1* (encoding to Snail) and *SNAI2* (encoding to Slug). (**e**) A549 cells were transfected with Myc or Myc-Pellino-1 (ΔC; 0, 1, 3, 6 *μ*g of plasmid). At 48 h post-transfection, the cells were harvested and subjected to immunoblotting for Myc, Snail, Slug and actin and (**f**) qRT-PCR analysis for *SNAI1* and *SNAI2*. (**g**) A549 cells were transfected with shLuc or shPellino-1 and subsequently treated with the proteasome inhibitor, MG132 (10 *μ*M) for 6 h. The cells were subsequently subjected to immunoblotting for Slug, Snail, Pellino-1 and actin. (**h**) A549 cells were transfected with GFP, GFP-Pellino-1 (FL, ring-like domain deletion (ΔC), ring-like domain (C)), shLuc, or 3′-UTR shPellino-1 plasmids. At 36 h post-transfection, the cells were harvested and immunoprecipitated with indicated antibodies. (**i**) A549 cells were transfected with Myc or Myc-Pellino-1. At 36 h post-transfection, the cells were treated with cycloheximide (CHX) for indicated times. The cell lysates obtained at each time point were subjected to immunoblotting for Snail, Slug, Myc and actin (top). The results were quantified through scanning densitometry of the Slug or Snail protein level with actin as an internal control (bottom). All data are shown as the means±S.D. of at least three independent experiments. The *P*-values were calculated using unpaired Student's *t*-test. *P <0.05; **P <0.01; ***P <0.005

The relationship between Pellino-1, Slug and Snail in lung cancer cells was further evaluated using human lung non-neoplastic and neoplastic cell lines. Pellino-1^Low^ BEAS-2B, H358 and PC9 cells were transfected with the Myc-Pellino-1, which resulted in an increase of Slug and Snail expression (Figure 5a). In contrast, Pellino-1^High^ H441, H1838, H1975 and HCC827 cells transfected with shPellino-1 or 3′-UTR shPellino-1 showed reduction of Slug and Snail expression (Figure 5b). Moreover, ectopic expression of Pellino-1 (WT) in A549 cells increased the expression of Snail and Slug in a dose-dependent manner (Figures 5c and d). However, ectopic expression of Pellino-1 (ΔC) in A549 cells had no effect on the expression of Snail and Slug (Figures 5e and f). These result indicated that Pellino-1 partially affects the transcriptional levels of Snail and Slug.

Next, treatment with a proteasome inhibitor (MG132) increased the expression of Slug and Snail in both shLuc and a shPellino-1-transfected A549 cell, suggesting that Slug and Snail protein stability might be regulated via a proteasome pathway (Figure 5g). In addition, we investigated whether overexpression of Pellino-1 in Pellino-1-depleted A549 cells rescue the expression of Snail and Slug proteins. Interestingly, overexpression of Pellino-1 wild-type (WT) recovered the levels of Slug and Snail proteins in Pellino-1-depleted A549 cells, but Pellino-1 mutants (ΔC and C domain) did not (Figure 5h). Taken together, these pull-down and immunoprecipitation assays revealed that Pellino-1 directly interacts and regulates Slug and Snail stabilization via FHA domain and E3 ligase activity.

Consistently, the degradation of Slug and Snail was delayed and their expression levels maintained in relatively steady-state levels in Pellino-1-overexpressing A549 cells treated with cycloheximide (inhibitor of protein synthesis), whereas Slug and Snail expression gradually decreased in control cells (Figure 5i). Taken together with aforementioned ubiquitination assay data, these results indicate that Pellino-1 stabilizes Snail and Slug protein via K63-mediated polyubiquitination.

Lung tissue from Pellino-1-Tg mice developing adenocarcinoma or related lesions showed Slug and Snail overexpression compared with lung tissue from non-Tg mice (Supplementary Figure S8), suggesting that Pellino-1 might affect the stability of Slug and Snail proteins *in vivo*.

### Slug and Snail are required for Pellino-1-mediated EMT and increased migration in lung cancer cells

**Figure 6:**
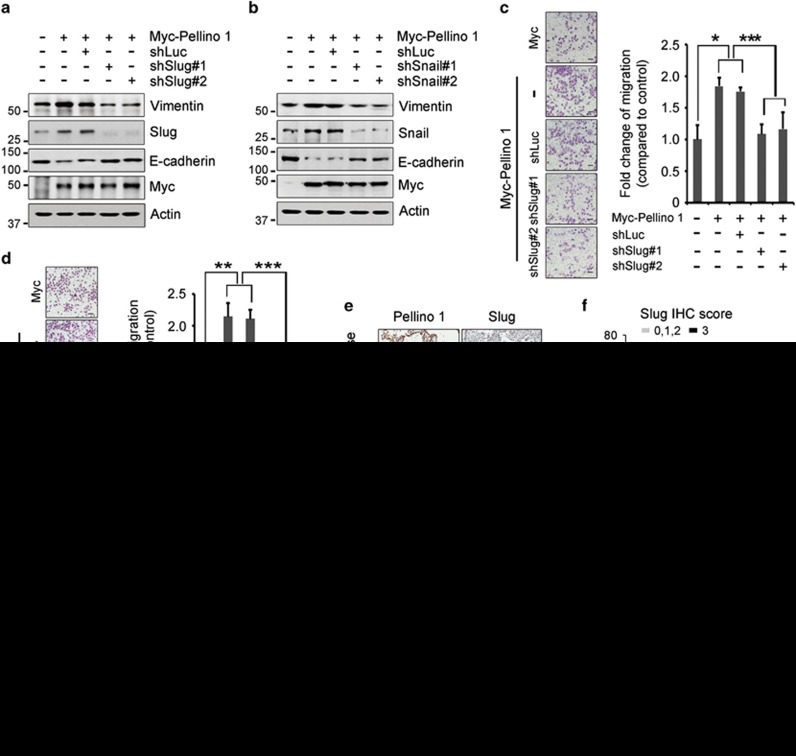
Slug and Snail are required for Pellino-1-mediated increased migration in A549 cells. Pellino-1 expression is correlated with Slug and Snail expression levels in human lung adenocarcinoma tissues. (**a** and **b**) A549 cells were transfected with Myc-Pellino-1 in combination with shLuc, shSlug#1, shSlug#2 (**a**) or shSnail#1, shSnail#2 (**b**). At 36 h post-transfection, the cells were harvested and subjected to immunoblotting for Myc, Slug, Snail, E-cadherin, Vimentin and actin. (**c** and **d**) A549 cells were transfected with Myc-Pellino-1 in combination with shLuc, shSlug#1, shSlug#2 (**c**) or shSnail#1, shSnail#2 (**d**) and subsequently subjected to transwell migration assay. Representative images are shown. The scale bars represent 100 *μ*m. The data are presented as the means±S.D. of at least three independent experiments. The *P*-values shown were calculated using unpaired Student's *t*-test. *P<0.05; **P<0.01; ***P<0.005. (**e**) Representative IHC images for Pellino-1 and Slug expression in lung adenocarcinoma patients who showed strong expression for both Pellino-1 and Slug (top) *versus* those showing no expression for both proteins (bottom). (**f**) The patients (*n*=169) were classified into two groups according to the following Pellino-1 and Slug expression levels: for Pellino-1, those with score 0 *versus* score 1–3, and for Slug, those with score 0–2 *versus* score 3. Patients with Pellino-1 expression showed higher Slug expression with statistical significance (*P*=0.016 by Pearson *χ*^2^ test). (**g**) Representative IHC images for Pellino-1 and Snail expression from lung adenocarcinoma patients who showed strong expression for both Pellino-1 and Snail (left) *versus* those showing little expression for both proteins (right) are shown. (**h**) A significantly strong positive correlation between the Pellino-1 and Snail IHC scores was observed (Spearman's *Rho*=0.445, *P*=0.000). (**i**) Patients (*n*=86) were classified into two groups according to the following Pellino-1 and Snail expression levels: for Pellino-1, those with score 0 *versus* score 1–3, and for Snail, those with score 0–1 *versus* score 2–3. Patients with Pellino-1 expression showed significantly higher Slug expression (*P*=0.000 by Pearson *χ*^2^ test)

To determine whether the expression of Slug and Snail was prerequisite for Pellino-1-mediated EMT phenotype, A549 cells were transfected with shSlug (shSlug#1 and shSlug#2) and shSnail (shSnail#1 and shSnail#2) (Supplementary Figure S9). The depletion of Slug and Snail upregulated E-cadherin expression in Pellino-1-overexpressing A549 cells (Figures 6a and b). In addition, Slug and Snail knock-down counteracted the increased migration observed in Pellino-1-overexpressing A549 cells (Figures 6c and d). These data indicated that Slug and Snail might have an important role in the Pellino-1-mediated EMT and increase in the migration of lung cancer cells.

### Correlation between Pellino-1, Slug and Snail expression levels in human lung adenocarcinoma tissues

Finally, the relationship between Pellino-1 and Slug or Snail expression was assessed in patients with lung adenocarcinoma. Representative immunohistochemistry (IHC) images for Pellino-1, Slug and Snail from patients who showed strong expression for both Pellino-1 and Slug or Snail, or those with little expression for both proteins are shown in Figures 6e and g. Pellino-1 expression (i.e., score 1–3) and strong Slug expression (i.e., score 3) showed statistically significant positive correlation (*P*=0.016 by Pearson *χ*^2^ test; Figure 6f). A strong positive correlation between Pellino-1 and Snail IHC scores was observed (Spearman's *Rho*=0.445, *P*=0.000; Figure 6h). Moreover, patients with Pellino-1 expression showed significantly higher Snail expression (score 2, 3) (*P*=0.000 by Pearson *χ*^2^ test; Figure 6i). Significant positive correlations between Pellino-1 and Slug or Snail expression were consistently observed in lung adenocarcinoma when separately analyzed according to the EGFR mutation status (Supplementary Figure S10). These results showed that Pellino-1 expression is positively correlated with Snail and Slug expression, particularly having a strong relationship with Snail, in human lung adenocarcinomas.

## Discussion

We observed that the overexpression of Pellino-1 promotes the development of a variety of lymphoid and solid tumors in Pellino-1-Tg mice.^30^ Thus, the deregulation of Pellino-1 is thought to have an oncogenic role in human cancer. However, the molecular mechanisms underlying the functional relevance of Pellino-1 in solid tumors, including lung cancer, has never been addressed. In this study, through *in vitro* and *in vivo* models using multiple lung cancer cell lines and xenograft and validation with human lung cancer tissues, we demonstrated that Pellino-1 has an oncogenic role in lung cancer through the stabilization of Snail and Slug via K63-mediated polyubiquitination, thereby promoting the EMT phenomenon. Pellino-1 was also revealed to promote cell proliferation and oncogenic transformation and activate Akt and ERK in lung cancer cells.

Various growth factors, such as epidermal growth factor (EGF), TGF-*β* and insulin-like growth factor-1 induced EMT in lung cancer and EMT was correlated with metastases and invasiveness of lung cancer.^17, 38, 39, 40, 41, 42^ This study provided a novel mechanism by which EMT is regulated in lung cancer. Pellino-1 was found to enhance cell proliferation and cell invasion and migration with the induction of EMT, as evidenced through cell morphology and the expression pattern of EMT-related markers. Pellino-1 induced EMT with the upregulation of Snail and Slug and downregulation of E-cadherin. The Snail and Slug, has a critical role in EMT, encoding transcriptional repressors in the E-cadherin promoter.^32, 33, 43^ Through a series of biochemical analysis, we here demonstrated that Pellino-1 directly interacts with Slug and Snail and increases the stability of these proteins via K63-mediated polyubiquitination, whereas Pellino-1 indirectly regulated E-cadherin.

Pellino-1 is an E3 ubiquitin ligase mediating K48- and K63-linked polyubiquitination, which destines the fate of target proteins into proteosomal degradation *versus* stabilization.^24, 44^ Moreover, recognition of substrate by Pellino protein is mediated by its forkhead-associated (FHA) domain, which binds to specific phosphothreonine motifs (i.e., pTxxD pTxxI/L pTxxS/A pTxxY/M), thereby endowing unique substrate specificities to Pellino proteins.^45^ Until now, a few numbers of Pellino's substrates with FHA-binding motif have been identified, including IL-1 receptor-associated kinase-1 (IRAK1), receptor-interacting protein-1 (RIP1), TNF receptor-associated Factor-6 (TRAF6), RIP2, cRel and BCL6.^30, 44, 45^ The presence of potential FHA-binding phosphothreonine motifs was found in Snail and Slug protein (Supplementary Table S2), which further support Snail and Slug as novel substrate of Pellino-1.

Snail and Slug overexpression was associated with aggressiveness, chemotherapy resistance and poor survival in patients with lung cancer. ^2, 17, 46^ This study showed that the overexpression of Pellino-1 is higher in lung adenocarcinoma rather than squamous cell carcinoma and other NSCLC histology. Moreover, Pellino-1 expression has a strong positive association with Snail or Slug expression in human lung adenocarcinoma. Together, these results indicate that Pellino-1 might function as an oncogene promoting EMT progress in human lung cancer, particularly in adenocarcinoma.

Pellino-1 is known as a critical molecule cascading IL-1R or TLR signaling and activating NF-κB and MAPK pathways during inflammatory and innate immune responses by mediating ubiquitination of signaling molecules.^27, 28, 29^ EGF promotes EMT through the upregulation of Snail and Slug and activates PI3K/Akt and ERK signaling pathways in lung cancer.^10, 17, 47^ Akt is a key regulator of NF-κB activation, and although initially implicated as distinct signaling pathways, the NF-κB and Akt signaling pathways often overlap.^48, 49^ Constitutive NF-κB activation was involved in EMT through the upregulation of EMT-inducing transcription factors in lung cancer cells.^50^ Lung cancer cells overexpressing activated mutants Akt underwent EMT along with Snail and Slug activation.^2, 12, 51^ ERK signaling also upregulated the expression of Snail and Slug in lung cancer.^17, 52^ In this study, Pellino-1 activated PI3K/Akt and ERK signaling, which was involved in upregulation of Slug and Snail and the downregulation of E-cadherin in A549 cells. These results suggested that Pellino-1 might promote EMT of lung cancer cells indirectly through PI3K/Akt and ERK activation as well as directly through the Slug and Snail stabilization. Moreover, Pellino-1 increased the phosphorylation (inactive form) of GSK3*β* in lung cancer cells. Given that active GSK3*β* promoted the degradation of Slug and Snail,^35, 36^ this study suggests that PI3K/Akt/GSK3*β* axis might also be involved in Pellino-1-mediated stabilization of Slug and Snail protein. However, the mechanism by which Pellino-1 activates PI3K/Akt and ERK pathway in lung cancer cells remains to be elucidated.

Meanwhile, the regulatory mechanism of Pellino-1 in lung cancer also remains elusive. Pellino-1 expression was higher in human pulmonary adenocarcinomas with EGFR mutation or increased gene copy number, and MET expression (Supplementary Table S1). However, there was no significant difference in the level of Pellino-1 between EGFR mutant and EGFR WT lung cancer cell lines (Figure 1d). EGF stimulation upregulated Pellino-1 expression in A549 cells, whereas EGFR inhibitor treatment decreased the Pellino-1 expression (Supplementary Figure S11). EGF-induced increase in cell migration and invasion were partly mediated by Pellino-1 (Supplementary Figure S11). Taken together, our data suggest that EGFR signaling might be involved in Pellino-1 upregulation in lung cancer but there is a possibility that another mechanisms are also involved. Thus, more studies are needed to clarify how Pellino-1 expression is regulated in lung cancer.

In summary, we provided the first evidence of a novel mechanism through which Pellino-1 contributes to lung tumorigenesis by promoting EMT through Snail and Slug K63-polyubiquitination and subsequent stabilization. These findings provide new insights into the role of Pellino-1 in carcinogenesis. Based on these results, we propose targeting the Pellino-1/Snail/Slug pathway as a potential therapeutic strategy to control lung cancer progression. Pellino-1 could be exploited for the development of effective therapeutic strategies for lung cancer.

## Materials and Methods

### Cell culture

Seventeen human lung cancer cell lines (A549, Calu-3, Calu-6, H322, H358, H441, H460, H1299, H1264, HCC1833, H1838, H1975, H820, PC9, H1650, HCC827, HCC4006; all adenocarcinoma except for H460, large cell carcinoma), non-neoplastic bronchial epithelial cell lines (BEAS-2B), lung fibroblastic cell lines (WI-26) and 293T cells were purchased from the American Type Culture Collection (Manassas, VA, USA). HCT116 cells were kindly gifted from Bert Vogelstein (Johns Hopkins Kimmel Cancer Center, Baltimore, MD, USA). BEAS-2B cells were maintained in bronchial epithelial cell growth medium (Lonza, Walkersville, MD, USA) and 10% FBS (Hyclone, Logan, UT, USA) supplemented with antibiotics. Other cell lines were grown in 90% RPMI-1640, DMEM or McCoy's medium and 10% FBS supplemented with antibiotics in a humidified 5% CO_2_ atmosphere.

### Plasmid construction and transfection

The FL cDNA sequence of the human Pellino-1 was PCR amplified using oligodT primers. Pellino-1-ΔC included 280 N-terminal amino acids and lacked the C-terminal RING domain. Pellino-1 FL, Pellino-1-ΔC and Pellino-1-C were subcloned into Myc-, GST-, GFP- or His6-tagged fusion plasmids. Small hairpin RNAs (shRNAs) targeting human Pellino-1 (shPellino-1), 3′-UTR Pellino-1 (3′-UTR shPellino-1), Slug (shSlug#1 and shSlug#2), Snail (shSnail#1 and shSnail#2) and the Luciferase (shLuc, as a control) were synthesis using the pSuper vector (Oligoengine, Seattle, WA, USA) according to gene-specific sequences described in Supplementary Materials and Methods. The pcDNA vector encoding Flag-tagged human GSK3*β* (Flag-GSK3*β*), Myc-tagged Pellino-2 (Myc-Pellino-2) and Myc-tagged Pellino-3 (α and *β*; Myc-Pellino-3α and Myc-Pellino-3*β*) was PCR amplified using oligodT primers. The pcDNA vector encoding Flag-tagged human Slug (Flag-Slug) and the pGEX-KG vector encoding GST-tagged human Slug (GST-Slug) were kindly gifted from Hong-Duck Um (Korea Institute of Radiological & Medical Sciences, Seoul, Korea). The pcDNA vector encoding Flag-tagged human Snail (Flag-Snail) and the pGEX-KG vector encoding GST-tagged human Slug (GST-Snail) were kindly gifted from Bum Joon Park (Pusan National University, Busan, Korea). HA-tagged ubiquitin K48 (HA-Ub K48), HA-tagged ubiquitin K63 (HA-Ub K63), the pEGFP-N3 vector encoding TAP (Strep-Flag-tagged) and His-tagged ubiquitin (His-Ub) were kindly gifted from Hong Tae Kim (Sungkyunkwan University, Suwon, Korea). For transient transfection, the cells were electroporated using a microporator (Digital Biotechnology, Seoul, Korea).

### Antibodies and reagents

The following antibodies were used: anti-Pellino-1 (F-7), anti-*β*-TrCP(H-85) (Santa Cruz Biotechnology, Santa Cruz, CA, USA), anti-actin, anti-Flag M2 (Sigma, St. Louis, MO, USA), anti-Slug, anti-Snail, anti-E-cadherin, anti-*β*-catenin, anti-Vimentin, anti-ERK1/2, anti-p-ERK1/2 (T202/Y204), anti-Akt, anti-p-Akt (S473), anti-GSK3*β*, anti-p-GSK3*β* (S9) (Cell Signaling Technology, Danvers, MA, USA), anti-HA, anti-Myc (Roche, Basel, Switzerland). The following reagents were used: LY294002, PD98059 (Calbiochem, San Diego, CA, USA), MG132, cycloheximide, dimethyl sulfoxide (DMSO) (AG Scientific, San Diego, CA, USA) and 3-(4,5-dimethylthiazol-2-yl)-2,5-diphenyltetrazolium bromide (MTT) (Sigma), EGF, mitomycin C (Sigma) and EGFR kinase inhibitor AG1478 (Selleckchem, Houston, TX).

### Cell proliferation, transwell migration and invasion assays, and colony-forming assay

Cell proliferation assay and colony-forming assay were performed as described in Supplementary Materials and Methods. Transwell migration assay was performed using uncoated cell culture inserts with 8 *μ*m pores (Corning incorporated, Tewksbury, MA, USA) and invasion assay was performed using cell invasion assay kits (Chemicon, Temecula, CA, USA), as described in Supplementary Materials and Methods.

### Western blot analysis

The cells were harvested and lysed in nuclear extraction (NE) buffer (20 mM HEPES (pH 7.6), 20% glycerol, 250 mM NaCl, 1.5 mM MgCl_2_, 0.1% Triton X-100, 1 mM PMSF, 1 mM DTT and protease inhibitor cocktail (Roche)). Equal amounts of protein were separated through SDS-PAGE and analyzed through immunoblotting with the indicated antibodies.

### *In vitro* binding and immunoprecipitation assays

For the S-tag pull-down assay, 293T cells were adapted to suspension conditions and lysed in NETN buffer (100 mM NaCl, 1 mM EDTA, 20 mM Tris-HCl (pH 8.0), 0.5% NP-40) containing 1 mM PMSF, 1 mM DTT and protease inhibitor cocktail. The supernatants were incubated with streptavidin-sepharose beads (Amersham Biosciences, Piscataway, NJ, USA) for 8 h at 4 °C and the bound proteins were analyzed via immunoblotting. For the GST pull-down assay, the fusion proteins were adsorbed onto glutathione-protein A/G Sepharose beads (Amersham Biosciences) and incubated with whole cellular extracts from A549 cells or purified His-tagged Pellino-1 proteins. The bound proteins were analyzed via immunoblotting. For immunoprecipitation, A549 cell pellets were resuspended in NETN buffer, incubated at 4 °C for 30 min and then lysed. The cell lysates were cleared by centrifugation and incubated with an anti-Pellino-1 antibody or normal IgG (control) and then with protein A/G agarose beads, which were later pelleted and analyzed by immunoblotting.

### *In vivo* ubiquitination assays

HCT116 cells were transfected with Myc, Myc-tagged Pellino-1-FL or Pellino-1-ΔC, Flag-Slug or Flag-Snail, and HA-tagged ubiquitin (HA-Ub), HA-Ub K48 or HA-Ub K63 plasmid in combination. At 48 h post-transfection, the cells were harvested and cell lysate were collected into two aliquots. One aliquot (10%) was used for conventional immunoblotting. The other aliquot (90%) were used for the immunoprecipitation with anti-Flag antibody and analyzed by immunoblotting.

### *In vivo* xenograft assay

To evaluate the effect of Pellino-1 on tumor growth, *in vivo* xenograft tumor model was used. Briefly, 6- to 8-week-old athymic nude (*nu*/*nu*) mice were housed in laminar-flow cabinets under specific pathogen-free conditions. A549 cells stably expressing shLuc, shPellino-1 or 3′-UTR shPellino-1 (5 × 10^6^ cells) were injected subcutaneously into the flank of mice (*n*=5 mice per group). Tumor size was measured every 3 days using caliper. Tumor volume in mm^3^ was calculated from the major (*a*) and minor (*b*) axis of the tumors using the following formula: V=*a* × *b*^2^/2. The animal studies and all procedures were approved by the Sungkyunkwan University School of Medicine Institutional Animal Care and Use Committee (IACUC).

### Immunohistochemistry for human lung cancer tissues

Formalin-fixed paraffin-embedded tumor tissues from patients with pulmonary adenocarcinoma (*n*=491), squamous cell carcinoma (*n*=59) and NSCLC of other histology (*n*=26) were collected. A tissue microarray with a 2-mm diameter was subjected to IHC. Clinicopathological data were retrieved from the medical records. This study was approved through the Institutional Review Board of Seoul National University Hospital (H-1211-049-440). IHC was performed using the Benchmark XT autostainer (Ventana Medical Systems, Tucson, AZ, USA) with antibodies against Pellino-1 (F-7) (Santa Cruz Biotechnology), Snail (LifeSpan BioSciences, Seattle, WA, USA), and Slug (Abcam, Cambridge, UK). The expression patterns were evaluated based on the intensity and proportion of staining in tumor cells and scored as follows: 0, negative or staining in <10% of tumor cells; 1, weak; 2, moderate; or 3, strong intensity in >10% of tumor cells. Statistical analyses were performed using SPSS software (version 21; IBM Corp., New York, NY, USA). Comparisons between variables were performed using the *χ*^2^ test or Spearman's correlation test, and two-sided *P*-values <0.05 were considered statistically significant.

### Quantitative real-time polymerase chain reaction (qRT-PCR)

Total RNAs were extracted from cells using RNeasy RNA extraction Mini kit (Qiagen Sciences, Germantown, MD, USA). The reverse transcription was carried out with an EasyScript™ cDNA Synthesis Kit (Applied Biological Materials Inc., Richmond, Canada) using Oligo (dT) primers. For qRT-PCR analyses, a Rotor-Gene Q real-time PCR detection system and SYBR® Green qPCR Master mixes (Qiagen Sciences) were used as follows: 95 °C for 5 min and 30 s, and 40 cycles (15 s at 95 °C, 1 min at 60 °C). The data were analyzed with a normalized gene expression method (ddCt) using Rotor-Gene Q software (Qiagen Sciences) and the *β*-actin were used as a reference for normalization. qRT-PCR was performed according to gene-specific sequences described in Supplementary Materials and Methods.

## Supplementary Material

Supplementary Informations
