# Supplementary material for: Pellino-1 promotes lung carcinogenesis via the stabilization of Slug and Snail through K63-mediated polyubiquitination
Source: Cell Death Differ. 2016 Dec 23;24(3):469–80. doi: 10.1038/cdd.2016.143 (PMC5457685; doi:10.1038/cdd.2016.143)
Supplement: Supplementary Informations [file cdd2016143x1.docx]

**Supplementary Information**

**Pellino-1 promotes lung carcinogenesis via the stabilization of Slug and Snail through K63-mediated polyubiquitination**

Yoon Kyung Jeon^1,5,6^ ,Chung Kwon Kim^2,5^, Kyung Rim Hwang^2^ , Hye-Young Park^2^, Jaemoon Koh^1, 3^, Doo Hyun Chung^1, 3^, Chang-Woo Lee^2, 4^ , and Geun-Hyoung Ha^2,6^

^1^Department of Pathology, Seoul National University Hospital, Seoul National University College of Medicine, Seoul 03080, Republic of Korea

^2^Department of Molecular Cell Biology, Samsung Biomedical Research Institute, Sungkyunkwan University School of Medicine, Suwon 16419, Gyeonggi-do, Republic of Korea

^3^Department of Biomedical Sciences, Seoul National University College of Medicine, Seoul 03080, Republic of Korea

^4^Samsung Advanced Institute for Health Sciences and Technology, Sungkyunkwan University, Suwon **06351**, Gyeonggi-do, Republic of Korea

^5^Co-first authors

^6^Co-corresponding authors.

**Supplementary Material and methods**

**Gene Sequences used for shRNA synthesis**

Gene-specific shRNA synthesis was performed using the following sequences: the Pellino-1-targeted small hairpin RNA (shPellino-1) 5’-GGGTTCAACACACTAGCAT-3’, 3’-untranslated region (UTR) Pellino-1-targeted shRNA (3’UTR shPellino-1) 5’-GCTCCTTTGGATATGCAATTT-3’, the human Slug shRNA (shSlug#1) 5’- CCCATTCTGATGTAAAGAAAT-3’, the human Slug shRNA (shSlug#2) 5’-TGCACATCCGAAGCCACAC-3’, the human Snail shRNA (shSnail#1) 5’- TGCACATCCGAAGCCACAC-3’, the human Snail shRNA (shSnail#2) 5’- GGAATATGTGAGCCTGGGCGCC-3’ and the Luciferase shRNA (shLuc, as a control) 5’-CTACGCGGAATACTTCGA-3’.

**Quantitative real time-polymerase chain reaction (qRT-PCR).**

Gene-specific primer for qRT-PCR was performed using the following sequences : E-cadherin 5’-aaggtgacagagcctctggat-3’ (forward) and 5’-cgtctgtggctgtgacct-3’; Snail 5’-ttctctaggccctggctgctacaa-3’ (forward) and 5’-tctctgacatctgagtgggtctgga-3’; Slug 5’-ctggtcaagaagcatttcaacgcc-3’ (forward)and 5’-aaagaggagagaggccattgggta-3’; β-actin 5’-ctacgtcgccctggacttcgagc-3’ (forward) and 5’-gatggagccgccgatccacacgg-3’ (reverse). β -catenin 5’-aaaatggcagtgcgtttag-3’ (forward) and 5’-tttgaaggcagtctgtcg-3’; Vimentin 5′-tgtccaaatcgatgatgtggatgaaac-3′ (forward) and 5′-ttgtaccattcttctgcctcctg-3′; Pellino 1 5’-gcaataagcaacaaag-3’ (forward) and 5’-atgagtcaaatcctgcag-3’ (reverse).

**Cell proliferation assay**

The cells were harvested from exponential growth phase and plated onto 96-well flat-bottomed microplates (200 μl cell suspensions, 2 × 10^4^ cells/ml). After the indicated time points, the cells were incubated with 2 mg/ml MTT for 3 hours and subsequently solubilized with DMSO. The optical density was spectrophotometrically measured at 490 nm.

**Transwell migration assay**

Transwell migration assay was performed using uncoated cell culture inserts with 8 µm pores (Corning incorporated, Tewksbury, MA, USA). The cells were harvested and suspended in 1 × 10^5^ cells per inserts in serum-free medium. The bottom chambers were filled with 750 µl of complete media. After incubation of 16 hours, the cells on the top surface of the filter were wiped off, and the cells that migrated to the bottom surface of the filter were fixed with 4% paraformaldehyde or ice-cold methanol, stained with 0.05% crystal violet for 10-20 minutes, washed and spectrophotometrically quantified at 490 nm.

**Invasion assay**

Invasion assay was performed using cell invasion assay kits (Chemicon, Temecula, CA, USA), according to the manufacturer’s instructions. The assay was done in an invasion chamber, consisting of a 24-well cell culture plate with 12 cell culture inserts. Cell suspension in serum-free media was added to the inserts, and each insert was placed in the lower chamber containing complete media. After 24 hours incubation, invasiveness was evaluated by staining of cells that had migrated through the extracellular matrix layer and clung to the polycarbonate membrane at the bottom of the insert.

**Colony forming assay**

The cells were harvested and suspended in 1 ml of top agar medium [RPMI containing 0.4% low melting agarose (Bio-Rad, Hercules, CA, USA) and 10% FBS] and quickly overlaid onto 2 ml of bottom agar medium (RPMI containing 0.8% low melting agarose and 10% FBS) in 6-well plates. After 2-3 weeks, the colonies were stained with 0.05% crystal violet and the numbers of colonies with a diameter larger than 100 μm were counted in four randomly selected microscopic fields per plate.

***In vivo* ubiquitination assays**

The in vivo ubiquitylation assay was conducted as described previously ^1^. A549 cells were transfected with TAP, TAP-Pellino-1, His-tagged ubiquitin (His-Ub) plasmid in combination. At 36 hours post-transfection, the cells were harvested, lysis with isolated through Ni-NTA beads (Qiagen). The bound proteins were immunoblotted with the indicated antibodies.

**Supplementary Tables**

**Table S1. Correlation between Pellino-1 expression and clinicopathologic features of pulmonary adenocarcinomas**

|  |  | Pellino-1 expression | | *P* | |
| --- | --- | --- | --- | --- | --- |
|  |  | Score 0, 1  n (%) | Score 2, 3  n (%) | |  |
| Age (yr) | ≤ 60 (n = 203) | 100 (49.3%) | 103 (50.7%) | | 0.014 |
|  | > 60 (n = 288) | 174 (60.4%) | 114 (39.6%) | |  |
| Size of tumor | ≤ 3 cm (n = 292) | 145 (49.7%) | 147 (50.3%) | | 0.001 |
|  | > 3 cm (n = 199) | 129 (64.8%) | 70 (35.2%) | |  |
| LN metastasis | Absent (n = 362) | 195 (53.9%) | 167 (46.1%) | | n.s. |
|  | Present (n = 129) | 79 (61.2%) | 50 (38.8%) | |  |
| Stage | 1 (n = 337) | 178 (52.8%) | 159 (47.2%) | | 0.044. |
|  | 2 (n = 46) | 33 (71.7%) | 13 (28.3%) | |  |
|  | 3 (n = 108) | 63 (58.3%) | 45 (41.7%) | |  |
| EGFR mutation | Wild-type (n = 168) | 110 (65.5%) | 58 (34.5%) | | 0.000 |
|  | Mutant (n = 226) | 104 (46%) | 122 (54%) | |  |
| EGFR FISH* | Negative (n = 134) | 98 (73.1%) | 36 (26.9%) | | 0.001 |
|  | Positive (n = 95) | 49 (51.6%) | 46 (48.4%) | |  |
| EGFR expression | Negative (n = 237) | 137 (57.8%) | 100 (42.2%) | | n.s. |
|  | Positive (n = 249) | 134 (53.8%) | 115 (46.2%) | |  |
| MET expression | Negative (n = 338) | 210 (32.1%) | 128 (37.9%) | | 0.000 |
|  | Positive (n = 153) | 64 (41.8%) | 89 (58.2%) | |  |
| ALK translocation | Absent (n = 469) | 263 (56.1%) | 206 (43.9%) | | n.s. |
|  | Present (n = 22) | 11 (50%) | 11 (50%) | |  |

*EGFR gene copy number was evaluated by FISH (fluorescence in situ hybridization) according to The University of Colorado Cancer Center (UCCC) criteria.

LN, lymph node; n.s., not significant

**Table S2.** **Prediction of FHA domain binding motifs in Pellino-1 substrates.**

| **Protein** | **Position** | **Sequence** | **+3 motif Protein** |
| --- | --- | --- | --- |
| **Snail** | 53 | ILNP**T**AS**L**P | **L** |
| **Snail** | 116 | SFSS**T**SV**S**S | **S** |
| **Snail** | 229 | AHLQ**T**HS**D**V | **D** |
| **Slug** | 26 | LDTH**T**VI**I**S | **I** |
| **Slug** | 234 | AHLQ**T**HS**D**V | **D** |

The FHA domain binding motifs of human Snail and Slug are listed. Positions refer to the amino acid number of the threonine shown in bold font. The amino acids at position +3 in the sequence are bolded and underlined according to the binding motif (pTxxD, pTxxI/L, pTxxS/A and pTxxY/M).

**Supplementary Figure Legends**

**Supplementary Figure S1.** Pellino-1 overexpression enhances the cell proliferation, migration and invasion in H1299 cells. **(a)** H1299 cells were transfected with Myc, Myc-tagged Pellino-1, shLuc, shPellino-1 or 3’UTR shPellino-1 and subjected to immunoblotting for Pellino-1 and Actin. **(b**) MTT assay was performed to estimate the cell proliferation in H1299 cells with Pellino-1 overexpression or knock-down. **(c-d)** H1299 cells with Pellino-1-overexpression or Pellino-1 depletion was subjected to transwell migration and invasion assays. The scale bars represent 100 μm. All data are shown as the means ± SD of at least three independent experiments. The *P* values were calculated using unpaired Student’s t test. *, *P* <0.05; **, *P* <0.01***, *P* <0.005.

**Supplementary Figure S2.** Pellino-1 overexpression enhances the cell proliferation, migration and invasion in non-neoplastic bronchial epithelial cell lines, BEAS-2B. **(a)** BEAS-2B cells were transfected with Myc, Myc-tagged Pellino-1 plus GFP vector (for transfection efficiency)**.** At 36 hours post-transfection, the cells were inspected for GFP expression under microscope (BF; bright field, *right*) and subjected to immunoblotting for Pellino-1, GFP and Actin (*left*). **(b)** MTT assay was performed to estimate the cell proliferation in BEAS-2B cells with Pellino-1 overexpression. **(c-d)** BEAS-2B cells with Pellino-1-overexpression or Pellino-1 depletion was subjected to transwell migration and invasion assays. The scale bars represent 100 μm. All data are shown as the means ± SD of at least three independent experiments. The *P* values were calculated using unpaired Student’s t test. *, *P* <0.05; **, *P* <0.01; ***, *P* <0.005.

**Supplementary Figure S3.** Pellino-2 and Pellino-3 (α and β) overexpression had no effect on the cell proliferation, migration and invasion in A549 cells. **(a)** A549 cells were transfected with Myc, Myc-tagged Pellino-2 or Pellino-3 (α and β). At 36 hours post-transfection, the cells were harvested and subjected to immunoblotting for Myc, and Actin antibodies. **(b)** MTT assay was performed to estimate the cell proliferation in A549 cells with Pellino-2 or Pellino-3 (α and β) expression. **(c-d)** A549 cells with Pellino-2 or Pellino-3 (α and β) overexpression was subjected to transwell migration and invasion assays. The scale bars represent 100 μm. All data are shown as the means ± SD of at least three independent experiments. The *P* values were calculated using unpaired Student’s t test. *, *P* <0.05; **, *P* <0.01; ***, *P* <0.005. (**e**) A549 cells were transfected with Myc, Myc-tagged Pellino-2 or Pellino-3 (α and β). At 36 hours post-transfection, the cells were harvested and subjected to immunoblotting for Myc, Snail, Slug, E-cadherin, and Actin antibodies.

**Supplementary Figure S4.** Pellino-1 directly interacts with GSK3β and inhibits GSK3β-mediated Slug and Snail degradation in A549 cells. **(a)** A549 cells were transfected with TAP (control), TAP-Pellino-1 (Flag-tagging Pellino-1). At 36 hours post-transfection, the cells were harvested and isolated through tandem affinity purification (TAP). The bound proteins were immunoblotted with GSK3β, β-Trcp1, Flag, and Actin antibodies. (**b**) A549 cells were transfected with Myc, Myc-Pellino-1 combination with Flag-GSK3β. At 36 hours post-transfection, the cells were harvested and subjected to immunoblotting with indicated antibodies.

**Supplementary Figure S5.** Pellino-1 interacts with Snail and Slug via FHA domain. (**a**) Schematic amino-acid sequence signatures of Pellino-1 plasmids. (**b**) A549 cells were transfected with GFP, GFP-Pellino-1 [Full length (FL), Ring-like domain deletion (ΔC), Ring-like domain (C)] plasmids. At 36 hours post-transfection, the cells were harvested and immunoprecipitated with an anti-GFP antibody. Pellino-1 protein complexes were subjected to immunoblotting with an anti-Snail, Slug, and GFP antibodies.

**Supplementary Figure S6.** E3 ligase activity of Pellino-1 enhances the migration in A549 cells. (**a-b**) A549 cells were transfected with Myc, Myc-Pellino-1-Full length (FL) or Myc-Pellino-1-∆C (ring domain deletion mutant). At 36 hours post-transfection, the cells were harvested and subjected to immunoblotting with indicated antibodies **(a)**. The results were quantified through scanning densitometry of the indicated protein level with actin as an internal control using Image J software **(b)**. **(c-d)** A549 cells were transfected with Myc, Myc-Pellino-1-FL or Myc-Pellino-1-∆C, and subsequently subjected to transwell migration assay. Representative images are shown (**c**). The scale bars represent 100 μm. The data are presented as the means ± SD of at least three independent experiments**.** The *P* values were calculated using unpaired Student’s t test. **, *P* <0.01; ***, *P* <0.005.

**Supplementary Figure S7.** Overexpression of Pellino-1 enhances the polyubiquitination of Snail and Slug in A549 cells. A549 cells were transfected with TAP, TAP-Pellino 1 combination with His-Ub plasmids. At 36 hours post-transfection, the cells were harvested, isolated through Ni-NTA-agarose. The bound proteins were immunoblotted with the indicated antibodies.

**Supplementary Figure S8.** Slug and Snail expression is higher in lung tissues of Pellino-1-Tg mice. Lung tissues from non-Tg and Pellino-1-Tg mice were lysed and subjected to immunoblotting for Pellino-1, Slug and Snail (**a**). The results were quantified through scanning densitometry of the Slug and Snail protein level using actin as internal control (**b**).

**Supplementary Figure S9.** Depletion of Snail or Slug reduced the cell proliferation in A549 cells. **(a)** A549 cells were transfected with shLuc, Snail-targeted shRNA (shSnail and 3’UTR shSnail) or Slug-targeted shRNA (shSlug & 3’UTR shSlug). At 36 hours post-transfection, the cells were harvested and subjected to immunoblotting for Slug, Snail, and Actin. **(b)** MTT assay was performed to estimate the cell proliferation in A549 cells with depletion of Snail and Slug at indicated times.. The *P* values were calculated using unpaired Student’s t test. *, *P* <0.05; **, *P* <0.01; ***, *P* <0.005.

**Supplementary Figure S10.** A significant correlation between Pellino-1 and Slug or Snail in human lung adenocarcinomas with or without EGFR mutation. (**a**) Patients with Pellino-1 expression showed significantly higher Slug expression with statistical significance among both EGFR wild-type group and EGFR mutant group (*P* = 0.041 and 0.047, respectively, by Pearson χ^2^ test). (**b**) Patients with Pellino-1 expression showed significantly higher Slug expression among both EGFR wild-type group and EGFR mutant group (*P* = 0.016 and 0.000, respectively, by Pearson χ^2^ test).

**Supplementary Figure S11.** EGFR signaling is involved in Pellino-1 overexpression in lung cancer. **(a)** A549 cells were treated with AG1478 (EGFR tyrosine kinase inhibitor) for 24 hours at indicated concentrations and harvested and subjected to immunoblotting for EGFR, Pellino-1 and Actin. **(b)** A549 cells were treated with EGF (100 ng/ml) and/or AG1478 (12.5 μM) for 24 hours and harvested and subjected to immunoblotting for indicated proteins. **(c)** A549 cells were treated with EGF (100 ng/ml) and/or AG1478 (12.5 μM) and subjected to transwell migration and invasion assay. **(d-e)** A549 cells transfected with shLuc or shPellino-1 were treated with EGF and subjected to immunoblotting for indicated proteins **(d)**, and transwell migration and invasion assay **(e)**. All data are shown as the means ± SD of at least three independent experiments. The *P* values were calculated using unpaired Student’s t test. *, *P* <0.05; **, *P* <0.01.

**References**

1. Xirodimas D, Saville MK, Edling C, Lane DP, Lain S. Different effects of p14ARF on the levels of ubiquitinated p53 and Mdm2 in vivo. *Oncogene* 2001, **20**(36)**:** 4972-4983.
